# Industrial Approach to Invertase Production from Fruit Waste for Enhanced Efficiency and Conservation

**DOI:** 10.1021/acsomega.4c01732

**Published:** 2024-06-05

**Authors:** Emre Dokuzparmak

**Affiliations:** Ege University, Department of Bioengineering, Faculty of Engineering, İzmir 35040, Turkey

## Abstract

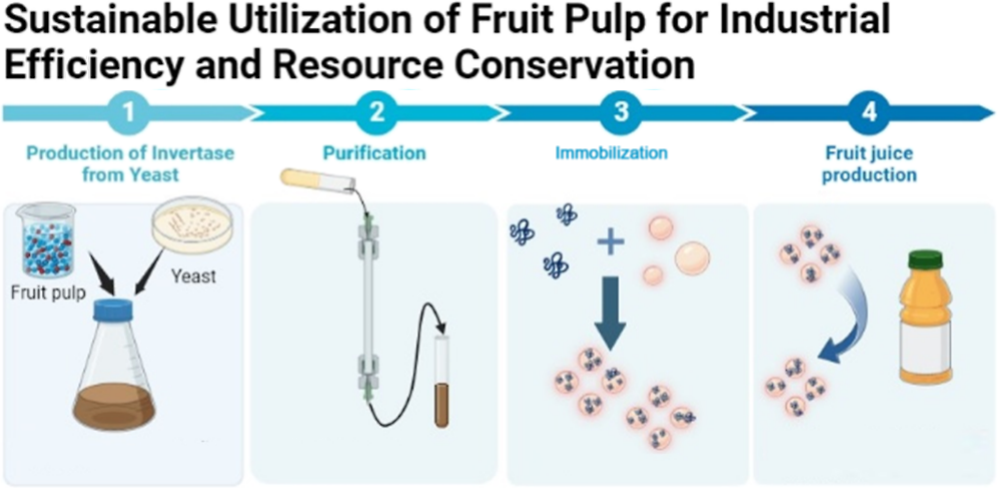

This study investigates the commercial viability of repurposing fruit waste for enzyme production, specifically focusing on the invertase enzyme derived from *Saccharomyces cerevisiae*. By utilizing fruit pulp that incorporates mulberry, carob, Figure, and grape pulp as a nutrient source, it is observed that the culture medium containing carob pulp exhibits the highest invertase activity. Specifically, the invertase activity in this medium is approximately 2.5 times greater (12.90 U/mg protein) than that observed in the peptone medium (5.98 U/mg protein). The extract undergoes several purification steps, including ultrafiltration, ammonium sulfate precipitation, dialysis, and ion-exchange chromatography (purification ratio: 12.11 times, yield: 26.93%). The purified enzyme is immobilized using alginate beads, improving pH and thermal stability. The immobilized enzyme exhibits optimal activity between pH 3.50 and pH 7.00, thereby broadening the enzyme’s high-activity pH range. The thermal stability of the immobilized invertase enzyme is significantly improved, especially at 65 °C. Activity studies in the presence of metal ions and certain chemicals have been conducted. The immobilized enzyme’s activity increases by approximately 40% in the presence of Ca^2+^ and Mg^2+^, and the immobilized enzyme maintains its activity in the presence of detergents such as SDS, Tween-20, and organic solvents like ethanol and methanol. The potential for the reuse of immobilized invertase was investigated under standard assay conditions. After 20 cycles, the immobilized enzyme was found to retain 80% of its initial activity. Overall, the study establishes the commercial potential of fruit pulp, typically discarded in fruit juice production, as a valuable source for obtaining an invertase enzyme. Furthermore, this study also aims to develop a suitable purification process for invertase in the fruit juice industry. By harnessing fruit waste and implementing innovative enzyme production strategies, industries can enhance their efficiency, reduce their environmental footprint, and optimize resource utilization.

## 1 Introduction

Enzymes are the most used type of macromolecules in our daily lives.^1^ They can be produced by various organisms and serve multiple functions in the body, ranging from intracellular signaling to their crucial role in energy production for humanity. Invertase (beta-fructofuranosidase) catalyzing the hydrolysis of sucrose is particularly important.^2^ It also acts as a hydrolyzing enzyme for other oligosaccharides, such as ketose, raffinose, and stachyose.^3^ Invertase is synthesized in plants, bacteria, fungi, and yeasts, and it can be extracted from these sources for industrial purposes.^4^ Invertase converts sucrose into invert sugar, which contains equal amounts of d-glucose and d-fructose.^5^ Especially, invertase from *Saccharomyces cerevisiae* is important in the food and beverage industry due to the yeast’s nonpathogenic and nontoxic properties.^6^ This enzyme has a homodimer structure with a molecular weight of 210 kDa.^7^ Post-translational modification steps are responsible for at least four isoforms of invertase, which exhibit different thermal and pH stability as well as different characteristics.^8^ Stability against heat and pH variations is crucial for industrial applications. The literature demonstrates that invertase isoforms exhibit varying chemical activities during industrial processes.^8,9^

The activity of an enzyme is a crucial factor that affects enzymatic reactions, and it can be influenced by various environmental factors such as substrate concentration, temperature, and pH.^14^ In industrial processes, there are several methods to preserve enzyme activity, including freezing, lyophilization, crystallization, and immobilization.^10^ İmmobilization can enhance the pH and thermal stability of an enzyme. There are several techniques for enzyme immobilization, such as adsorption, covalent bonding, entrapment, cross-linking, and encapsulation.^11^ The immobilization of enzymes onto inert or insoluble materials such as alginate gel can provide more resistance to changes in process conditions such as pH and temperature. Moreover, immobilized enzymes can be easily separated from the products and reused during the process.^10,11^

Fruit pulp generally contains high levels of moisture, carbohydrates, and minerals such as calcium, magnesium, potassium, and phosphorus.^12^ It also contains a significant amount of protein and fat, as well as antioxidants, natural coloring agents, and bioactive compounds in some cases.^15^ Due to the quantity and biochemical properties of fruit pulp obtained from industrial processes, several studies have been conducted on its potential use as a nutrient material in industry, particularly for products such as enzymes, organic acids, and sweeteners.^16^

Fruit pulp, often seen as waste in the production of fruit juice, is a resource with a wide range of uses. It is commonly used in the food industry, especially in making jams, jellies, and desserts based on fruits.^17^ Additionally, due to its high content of dietary fibers, vitamins, and minerals, fruit pulp is an essential part of a nutritious diet. In recent times, there has been an increase in the use of fruit pulp in biotechnological applications. For example, enzymes, which are valuable biochemical substances, can be obtained from waste fruit pulp. This is crucial for both the valorization of waste and the advancement of sustainable biotechnological processes. Hence, fruit pulp holds significant value for the food and biotechnology sectors.^17^

Emerging as a global force in fruit production, Turkey witnessed a remarkable surge in its fruit yield from 2018 to 2023. This period of growth culminated in 2023, with a record harvest exceeding 27.4 million metric tons of fruit. A part of this large fruit production is converted into fruit pulp, which finds many uses in various industries.^18^ Consequently, the composition of fruit pulp was characterized and examined. Mulberry pulp is characterized by its high content of carbohydrates (7.8–9.0%), protein (0.5–1.4%), fatty acids (0.3–0.5%), free acid (1.1–1.8%), fiber (0.9–1.3%), and ash (0.8–1.0%), along with a moisture content of 85–88%. It also notably contains arabinose (20.29%) and xylose (6%), among other sugars.^19^ Carob pulp is a mixture of both macro- and micronutrients. This includes carbohydrates, vitamins, minerals, and secondary metabolites, all of which possess beneficial properties. It is primarily composed of carbohydrates (mainly sucrose), fibers, minerals, vitamins, and a significant amount of protein, while maintaining a low-fat content.^20^ The pulp of Figures is composed of various compounds, such as sugar, water, aromas, potassium, tartaric acid, and malic acid. However, it is worth noting that the total phenol, total flavonoid, and total anthocyanins of the frozen Figures decrease significantly compared to fresh Figures.^21^ Lastly, grape pulp, which is the heaviest and most voluminous part of the grape, contributes the most material during the crushing phase. Its main components are water, sugars, acids, mineral salts, and vitamins. It also contains a variety of compounds including sugar, water, aromas, potassium, tartaric acid, and malic acid.^22^

Ion-exchange chromatography is a widely employed technique in the purification of industrial enzymes.^23,24^ This approach leverages the varying surface charges of proteins and is frequently used to track the deamidation and succinimide formation. Anion-exchange chromatography is carried out at pH levels higher than the enzyme’s isoelectric point, while cation-exchange chromatography occurs below the isoelectric point, attracting positively charged enzymes. The protein of interest can be gathered simply by adjusting the pH of the elution buffer.^23,24^ Hence, ion-exchange chromatography is highly appropriate for use in industrial enzyme purification processes. However, the selection of the most suitable method for a particular application hinges on the target protein’s characteristics, the requirements for purity, and other considerations.^24^

In this study, four different yeast strains (*S. cerevisiae*) were utilized. These strains are known for their high levels of invertase production. The research aims to enhance yeast growth conditions by incorporating products such as fruit pulp into the growth medium, thereby increasing invertase activity and achieving higher yields. The study demonstrates the potential of industrial fruit pulp as a nutrient source in the growth environment. Additionally, the most effective growth medium with the highest invertase activity was optimized by adding fruit pulp. The invertase enzyme obtained was purified by using chromatography techniques such as ion-exchange chromatography. Subsequently, the purified enzyme was immobilized within alginate beads by using encapsulation. This approach was designed to enhance enzyme stability against pH and temperature variations. The goal was to facilitate the hydrolysis of sucrose in fruit juice by utilizing the immobilized enzyme within the alginate beads. Notably, this study introduces an innovative approach to enhance invertase activity during yeast growth using fruit waste. This not only provides a sustainable alternative to traditional sources like molasses but also contributes to minimizing environmental impacts by re-evaluating fruit waste.

## 2 Materials and Methods

### 2.1 Materials

All chemicals used were of analytical grade and were purchased from Sigma-Aldrich. Type-1 (yeast––YSC1––*S. cerevisiae*) and Type-2 (yeast––YSC2––*S. cerevisiae*) were acquired from Sigma-Aldrich, while Type-3 (instant dry yeast––*S. cerevisiae*) and Type-4 (fresh/wet yeast––*S. cerevisiae*) were obtained from the market. MgSO_4_, (NH_4_)_2_SO_4_, and K_2_HPO_4_ were purchased from Merck Chemicals. Glucose, peptone, dextrose, and yeast extract were purchased from Sigma-Aldrich. SDS-PAGE Sample Prep Kit was obtained from Thermo Fisher Scientific. Fruit pulps (mulberry, carob, Figure, and grape pulp) were obtained from fruit juice factories in Turkey.

### 2.2 Yeast Strain, Culture Conditions, and Preparation of the Enzyme Extract

Four different industrial strains of Baker’s yeast *S. cerevisiae* (the live and dried yeast obtained from the market) were incubated in a shaker incubator at 37 °C for 24 h in a yeast extract peptone dextrose (YEPD) medium containing fruit pulp, 20 g/L glucose, 20 g/L peptone, and 10 g/L yeast extract, derived from *S. cerevisiae* strains. *S. cerevisiae* yeast was initially incubated in 50 mL volumes, procured from a stock maintained at −40 °C. Following this, a scale-up was performed to a volume of 1000 mL [5% (v/v)] to facilitate large-scale production. The mature seed culture [5% (v/v)] was inoculated into different types of fermentation medium as provided in Table 2, and the culture was incubated in a shaker incubator at different temperatures and durations specified in Table 2. A 50 mM acetate buffer was used to adjust the pH. To collect the supernatant used for invertase activity, the culture was centrifuged at 4 °C and at 4000 rpm for 20 min.

### 2.3 Purification of Invertase

The ammonium sulfate precipitation method was used to precipitate proteins in the crude extract. The gradual addition of ammonium sulfate from 10 to 90% was used to take fractions from the supernatant. After centrifugation at 20,000 rpm for 20 min at 4 °C, the precipitates were resuspended in 50 mM (pH 5.0) sodium acetate buffer and placed in a dialysis bag (MWCO 12,000 Da) in the same buffer. The medium was desalted from the remaining ammonium sulfate and loaded onto a QAE Sephadex A50 ion-exchange column (2 × 40 cm). A linear gradient of NaCl (0 to 1 M) was established in 50 mM of sodium acetate buffer (pH 5.0), and elution was carried out by adjusting the flow rate of the system to 3.5 mL/min. The presence of proteins in the collected fractions was monitored by measuring absorbance at 280 nm (SHIMADZU, UV-1900i), and the active fractions were collected and concentrated by ultrafiltration (Ultra cell Membrane 10,000 MWCO Millipore). The protein concentration was determined using the Bradford method, with bovine serum albumin as the standard.^25^ A calibration curve was plotted at 595 nm with the values obtained. The protein purity was then determined by SDS-PAGE (sodium dodecyl sulfate-polyacrylamide gel electrophoresis) analysis according to the literature.^26^

### 2.4 Polyacrylamide Gel Electrophoresis (SDS-PAGE)

The SDS-PAGE analysis was performed on a vertical system, specifically the VWR Vertical PAGE System, using a stacking gel (6% polyacrylamide gel at pH 6.8) and a separation gel (12% polyacrylamide gel at pH 8.8). Tris buffer with 0.1% sodium dodecyl sulfate was utilized in the SDS-PAGE system, while 0.1% of Coomassie brilliant blue R-250 was applied for staining the gels.^26^ To determine the molecular weight of the target protein, a comparison was made to marker proteins.

### 2.5 Invertase Activity and Concentration Determination

A volume of 25 μL of invertase was introduced into a mixture consisting of 0.3 M sucrose and 50 mM acetate buffer (475 μL) at a pH of 4.5. After a reaction time of 5 min at 25 °C, the reaction was stopped by adding 2,4-dinitrosalicylic acid (DNS) reagent (500 μL), and the mixture was then boiled in a water bath for 5 min. The absorbance at 540 nm was measured after adding 4 mL of deionized water.^27^ The standard curve was created by employing different concentrations of an equimolar mixture of d-glucose and d-fructose in 50 mM acetate buffer with a pH of 4.5, ranging from 0.5 to 10 mM. One unit of invertase activity (U) is defined as the quantity of enzyme that catalyzes the hydrolysis of 1 μmol of sucrose in 1 min under the given assay conditions. The concentration of the invertase was determined by measuring the absorbance at 280 nm.^28^

### 2.6 Immobilization of the Purified Invertase Enzyme

The process of immobilizing the purified invertase involved encapsulation within calcium alginate. To achieve this, a mixture of aqueous sodium alginate [2% (w/v)] and invertase was extruded through a pipet into a CaCl_2_ solution, resulting in the formation of calcium alginate beads that entrapped invertase. The beads were collected by filtration and then washed with distilled water to remove any excess CaCl_2_ and nontrapped enzyme. The resulting capsules were dried between two sheets of filter paper and in open air for 2 h prior to use. The binding percentage was calculated by subtracting the remaining enzyme content in the filtrate solution from the initial enzyme concentration.^29^ The immobilization yield was determined by using eq 1.
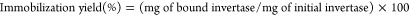
1

### 2.7 Optimum pH and Stability

To determine the optimal pH range, assays were conducted for both free and immobilized invertase using McIlvaine buffer at pH 2.20 to 8.00 and glycine–NaOH buffer at pH 9.00 to 11.00. The highest activity was considered as 100%, and relative activity was calculated accordingly. The pH stability of the free enzyme was evaluated by mixing it with 50 mM McIlvaine buffer and 50 mM glycine–NaOH buffer for pH 2.5, 4.0, 6.0, and pH 8.0. The mixture was then assayed for activity after being stored at room temperature for 120 h. Residual activity was determined by comparing it to the initial enzyme activity. The pH stability of the immobilized enzyme was also measured by mixing it with buffer solutions at pH 2, 4, 6, and 8 and storing it at room temperature for up to 120 h. For the immobilized enzyme, 0.5 g of solid support (alginate beads) was mixed with 1 mL of the buffer for the pH stability of the immobilized enzyme. Activity assays were carried out, and the residual activity was calculated by comparing it with the initial enzyme activity.

### 2.8 Optimum Temperature and Thermal Stability

The optimal temperature for the free enzyme was established by performing activity assays over a temperature range from 25 to 80 °C. In a similar manner, the optimal temperature for the immobilized enzyme was determined by conducting activity assays within the same temperature span. The results were calculated as relative activity, with the highest activity considered as 100%. To determine the thermal stability of the free enzyme, the enzyme solution was incubated at 4, 25, 37, 65, and 85 °C in Eppendorf tubes, and aliquots were examined for 120 h.

The aliquots taken were quickly cooled to room temperature, and enzyme activities were determined under the standard conditions. For the immobilized enzyme, 0.5 g of solid support was mixed with 1 mL of pH 9.00 and 10.00 buffers separately for the alginate-immobilized enzyme, respectively. The thermal stability of the immobilized enzyme was determined by incubating these mixtures for up to 120 h from 4 to 85 °C. The results were calculated as residual activity by comparing with nonincubated enzyme activities.

### 2.9 Determination of the Kinetic Constant

The industrial performance of the immobilized invertase enzyme was elucidated. Enzyme kinetic studies were conducted with a focus on the *K*_m_ and *V*_max_ values. These parameters represent the enzyme’s affinity for its substrate and the maximum reaction speed, respectively. The reaction rate was determined at different sucrose concentrations for both free and immobilized enzymes. *K*_m_ is calculated for the immobilized invertase using sucrose substrate. Sucrose concentrations were varied within the range of 2.5 to 300 mM. A Lineweaver–Burk plot was utilized for performance comparison.

### 2.10 Reusability

Following each hydrolysis process, the calcium alginate beads were collected and thoroughly washed with distilled water before being stored at 4 °C for subsequent use. This procedure was repeated for 25 cycles. The catalytic efficiency of the immobilized enzyme was evaluated over a period with a 2 h time interval between measurements.

### 2.11 Effect of Some Chemicals on the Enzyme Activity

The impact of metal ions on the enzyme activity was assessed by adding chloride salt solutions of Na^+^, K^+^, Li^+^, Al^3+^, Fe^3+^, Ca^2+^, Co^2+^, Cu^2+^, Fe^2+^, Mg^2+^, Mn^2+^, Ni^2+^, and Zn^2+^ ions at the final concentrations of 1, 5, and 10 mM. The impact of SDS, ethanol, methanol, acetone, 2-propanol, EDTA, chloroform, and Tween 20 at the final concentrations of 1, 5, and 10% (v/v) was also investigated. For residual activity estimations, enzyme activity was determined in the absence of chemicals, and the activity observed in this condition was defined as 100%.^30^

### 2.12 Industrial Efficiency of Immobilized Invertase by HPLC

A Shimadzu, Nexera-i, LC-2040C 3D Model evaporative light-scattering detector (ELSD LT-II Model) was used for the analyses. Chromatographic separations were performed on a Phenomenex Luna NH2 column (5 μm particle size, 250 × 4.6 mm id, 100 Å) with an isocratic elution of ACN/H_2_O (80:20, v/v). The column oven temperature was set at 40 °C, and the injection volume was 10 μL. The mobile phase was pumped through the HPLC–ELSD system at a flow rate of 1.5 mL/min. Each run was conducted within 30 min.

## 3 Results and Discussion

Invertase was isolated from four different industrial strains of Baker’s yeast *S. cerevisiae*, using the extraction process mentioned in the Materials and Methods section. The invertase activity was detected by the DNS method.^27^ These preparations were analyzed, and here-presented results showed that the invertase from yeast Type-1 has the highest activity (Table 1). Additionally, the invertase enzyme from Type-1 was purified and immobilized.

**Table 1:** Activities of Invertase in the Crude Extract of Four Types of Yeasts Incubated at 37 °C for 24 h in YEPD^a^

	type-1	type-2	type-3	type-4
activity (U/mg protein)	12.71 ± 0.86	9.31 ± 1.08	6.84 ± 0.67	8.15 ± 0.88

a To collect the supernatant used for invertase activity, the culture was centrifuged at 4 °C and at 4000 rpm for 20 min.

The Type-1 strain, which exhibited the highest invertase activity, was selected and tested to determine the optimal conditions for enhanced invertase production. Experiments were conducted to determine the optimal growth conditions for Type-1. For that purpose, 12 different growth media were prepared in the presence of different temperatures, pHs, and incubation periods (Table 2). Type-1 was incubated in YEPD at 37 °C for 24 h. To collect the supernatant, the culture was centrifuged at 4 °C and at 4000 rpm for 20 min. The invertase activity was detected by the DNS method.^27^ The growth medium that demonstrated the highest invertase activity (T6) was selected as the optimal growth medium for subsequent experiments.

**Table 2:** Determination of the Optimum Medium Conditions for Type-1 in YEPD with Varying Temperatures (30–60 °C), pH’s (4.0 and 8.0), and Incubation Periods (24–96 h)

medium code	incubation period (h)	temperature (°C)	pH	activity (U/mg protein)
T1	24	30	4	7.45 ± 0.75
T2	48	30	4	9.65 ± 1.12
T3	72	30	4	6.91 ± 1.07
T4	96	30	4	4.47 ± 0.61
T5	48	30	5	10.02 ± 0.73
T6	48	30	6	12.88 ± 0.98
T7	48	30	7	11.61 ± 1.24
T8	48	30	8	10.94 ± 0.81
T9	48	20	5	9.03 ± 0.64
T10	48	40	5	8.47 ± 0.55
T11	48	50	5	3.66 ± 0.91
T12	48	60	5	1.14 ± 1.52

To induce higher amounts of invertase enzyme from this strain, 13 different culture media were prepared, containing peptone, different sugars, and industrial waste materials such as mulberry, carob, Figure, and grape pulp (Table 3). The industrial fruit pulp was added to these media to obtain high amounts of invertase enzyme. M1 (YEPD medium) was considered the standard culture medium, and the other culture media were compared to the standard medium.

**Table 3:** Different Growth Media for Type-1 Yeast in the Presence of Sugar Derivatives, Molasses, MgSO_4_, (NH_4_)_2_SO_4_, K_2_HPO_4_, and Fruit Pulp (Carob, Mulberry, and Grape Pulp) Under the Optimum Conditions (at 30 °C, at pH 6.0 for 48 h)

medium code	medium components	composition (g/L)	activity (U/mg protein)
M1	peptone	20	5.98 ± 1.24
	dextrose	20	
	yeast extract	10	
M2	peptone	20	8.15 ± 0.78
	sucrose	20	
	glucose	20	
	MgSO_4_	1	
	(NH_4_)_2_SO_4_	1	
	K_2_HPO_4_	1,7	
	yeast extract	10	
M3	peptone	40	7.77 ± 1.01
	sucrose	20	
	glucose	20	
	MgSO_4_	1	
	(NH_4_)_2_SO_4_	1	
	K_2_HPO_4_	1,7	
	yeast extract	10	
M4	peptone	20	6.76 ± 0.68
	sucrose	40	
	glucose	20	
	MgSO_4_	1	
	(NH_4_)_2_SO_4_	1	
	K_2_HPO_4_	1,7	
	yeast extract	10	
M5	peptone	20	10.05 ± 0.87
	sucrose	20	
	MgSO_4_	1	
	(NH_4_)_2_SO_4_	1	
	K_2_HPO_4_	1,7	
	yeast extract	10	
M6	peptone	20	1.19 ± 1.82
	dextrose	20	
	beef extract	10	
M7	peptone	20	0.83 ± 1.71
	sucrose	20	
	glucose	20	
	NaCl	3	
	sugar molasses	20	
M8	peptone	30	1.29 ± 1.42
	NaCl	2	
	sugar molasses	30	
M9	peptone	20	12.8 ± 0.77
	sucrose	20	
	MgSO_4_	1	
	(NH_4_)_2_SO_4_	1	
	K_2_HPO_4_	1,7	
	yeast extract	10	
	carob pulp	20	
M10	peptone	20	10.3 ± 0.83
	sucrose	20	
	MgSO_4_	1	
	(NH4)2SO4	1	
	K_2_HPO_4_	1,7	
	yeast extract	10	
	mulberry pulp	20	
M11	peptone	20	8.26 ± 0.56
	sucrose	20	
	MgSO_4_	1	
	(NH4)2SO4	1	
	K_2_HPO_4_	1,7	
	yeast extract	10	
	grape pulp	20	
M12	peptone	20	11.9 ± 0.99
	sucrose	20	
	MgSO_4_	1	
	(NH4)2SO4	1	
	K_2_HPO_4_	1,7	
	yeast extract	10	
	figure pulp	20	
M13	sugar molasses	20	3.27 ± 1.15
	dextrose	10	
	yeast extract	10	

Research in the literature has shown that certain metal ions, specifically calcium (Ca^2+^), potassium (K^+^), and magnesium (Mg^2+^), have an effect on the activity of the invertase enzyme.^31,32^ M1 is a YEPD medium. M2 was prepared as a comparison medium to distinguish the effect of the presence of metal ions from the effect of using fruit pulp. Furthermore, the impact of additional components such as peptone, sucrose, and glucose on invertase activity was also investigated, leading to establishment media M2, M3, M4, M5, and M6 for comparative analysis. Also, invertase media with fruit pulps (M9, M10, M11, and M12) were prepared. This method made it easier to distinguish between the effects caused by the fruit pulp and those caused by the metal ions. After the results were examined, it was clear that the medium with fruit pulp showed the most significant activity of the invertase enzyme. Additionally, M13 medium was prepared for the purpose of comparing the effect of molasses with that of the fruit pulp medium.

As shown in Table 3, the media containing a combination of MgSO_4_, (NH_4_)_2_SO_4_, K_2_HPO_4_, and fruit pulp (carob pulp (M9) and Figure pulp (M12)) showed higher invertase activity. In addition, the absence of MgSO_4_, (NH_4_)_2_SO_4_, and K_2_HPO_4_ in media (M1) resulted in low invertase activity. The medium containing carob pulp (M9) had the highest invertase activity (12.8 ± 0.77 U/mg protein), which was approximately 50% higher than that of the invertase activity (8.15 ± 0.78 U/mg protein) in M2 medium. The medium containing Figure pulp (M12) also showed the second highest invertase activity (11.9 ± 0.99 U/mg protein). The results showed that the presence of industrial waste materials increased the invertase activity, indicating the potential of these waste materials to be used as a contribution to invertase production processes and gain commercial value.

### 3.1 Purification of Invertase

An extract of invertase was obtained through the autolysis of yeast cells. The extract was then purified through multiple steps including ultrafiltration, ammonium sulfate precipitation, dialysis, and ion-exchange chromatography. The results of all purification steps are summarized in Table 4.

**Table 4:** Purification Steps of Invertase and Yield (%) for Each Purification Step

	activity (U/ml)	specific activity (U/mg)	purification ratio	yield (%)
crude extract	2784.96 ± 81.17	0.88 ± 0.42	1	100
ultrafiltration	836.93 ± 65.14	3.41 ± 0.94	3.86	30.05
ammonium sulfate	787.70 ± 53.89	6.15 ± 0.68	6.96	28.28
dialysis	777.08 ± 32.16	6.69 ± 0.37	7.57	27.90
QAE-Sephadex A50	750.05 ± 37.42	10.71 ± 0.51	12.11	26.93

**Figure 1:**
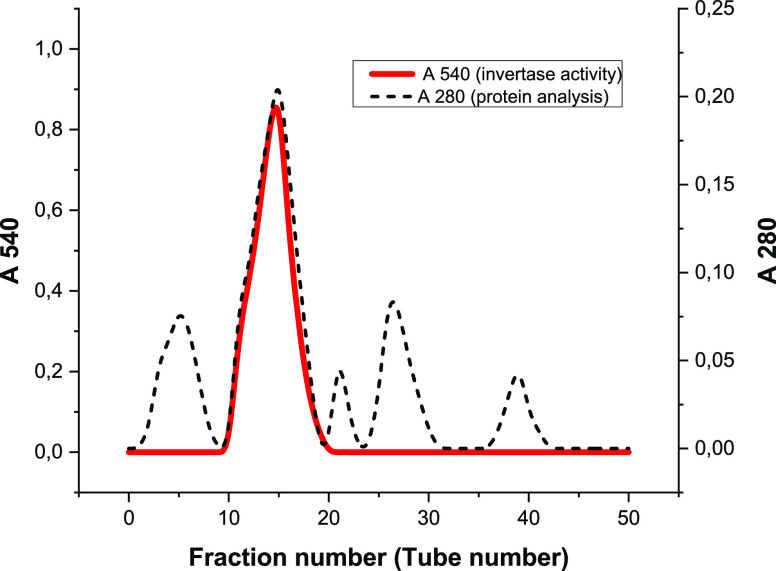
Protein analysis at 280 nm and invertase activity results at 540 nm for fractions (2 mL) collected after QAE-Sephadex ion-exchange column chromatography in the presence of a NaCl salt bridge with a linear gradient from 0 to 1 M.

Fractions exhibiting the highest protein content and invertase activity were collected and used to obtain the purified invertase enzyme (Figure 1). These fractions were subsequently used as purified invertase enzyme. After all purification processes, the enzyme was purified approximately 12-fold from the crude extract with 26.93% yield. The purified invertase exhibits a high specific activity of 10.71 U/mg of protein (Table 4).

El-Ghonemy et al. identified β-d-fructofuranosidase from a novel fungus.^33^ The enzyme was purified through ammonium sulfate salt fractionation, ion-exchange chromatography on DEAE-cellulose, and Sephadex G-100 gel filtration, resulting in a 13.3-fold purification rate, 22.6% yield, and 192.9 U/mg protein specific activity. The optimal pH and temperature were determined to be 6.0 and 50 °C, respectively. It has been highlighted that Aspergillus sp. DHE1 β-d-fructofuranosidase may have numerous applications in the food industry. In another investigation, the extraction of β-d-fructofuranosidase was carried out from Fusarium solani and resulted in a threefold purification with a yield of 9.33%.^34^ A study focused on purifying invertase from Aspergillus phoenicis utilized ion-exchange column chromatography (DEAE cellulose) and Sephacryl S-200, resulting in a 14.46% yield and 18.77 purification fold.^35^ However, purification of β-d-fructofuranosidase through numerous purification stages led to lower yields and was found to be a time-consuming and expensive process.^36^ Compared to the literature, our study has shown positive results in terms of cost, time, and purification efficiency (purification ratio: 12.11 times and yield: 26.93%) with a single-column purification step.

### 3.2 Electrophoretic Analysis of Invertase

**Figure 2:**
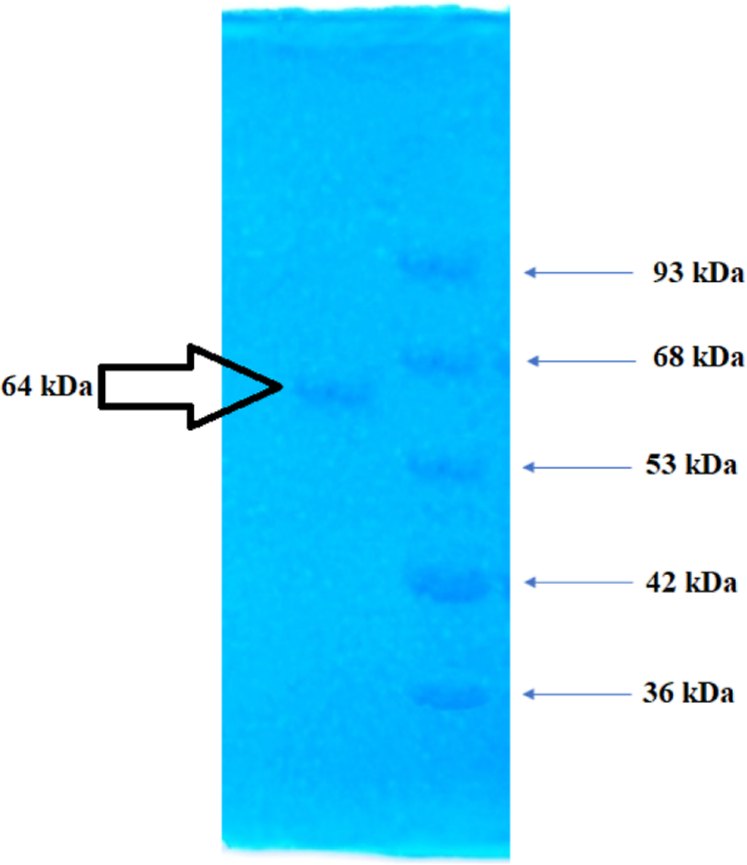
Electrophoretic profile of the invertase from Type-1; arrows indicate the position of the markers.

Purified invertase from Type-1 was displayed at a single band on SDS-PAGE gel electrophoresis (Figure 2). The molecular weight of the purified invertase was estimated as 64 kDa. The value of the single band observed by SDS-PAGE was compared with the kDa values of the standard markers. For this purpose, a standard graph was plotted using the *R*_f_ values of the standard markers. The molecular weight of the invertase enzyme was calculated in kDa using its *R*_f_ values and the standard graph.

In SDS-PAGE studies conducted on invertase isoforms from similar sources, it has been observed that invertase has a characteristic diffusion band at 60 kDa values.^8^ The presence of a single band on SDS-PAGE indicates that the invertase has been highly purified using ion-exchange chromatography, and the observation of this single peak at 64 kDa confirms that the invertase has been successfully purified from the optimized growth medium.

### 3.3 Immobilization of the Invertase

The purified invertase enzyme was immobilized by encapsulation within calcium alginate beads. To achieve this, a mixture of sodium alginate [2% (w/v)] and the enzyme was extruded into a CaCl_2_ solution [2% (w/v)], forming the beads. The resulting capsules were dried, and the binding percentage was calculated. As a result, encapsulation of invertase enzyme into alginate beads was performed in the presence of a glutaraldehyde cross-linker. The enzyme binding was determined as 42% based on activity analyses performed after immobilization. The *V*_max_ and *K*_m_ values of the invertase enzyme showed a slight increase after immobilization. The *V*_max_ value increased from 434.78 U/mg (pure enzyme) to 513.97 U/mg (immobilized enzyme), and the *K*_m_ values were determined as 1.27 mM for the pure enzyme and 1.54 mM for the immobilized enzyme.

**Figure 3:**
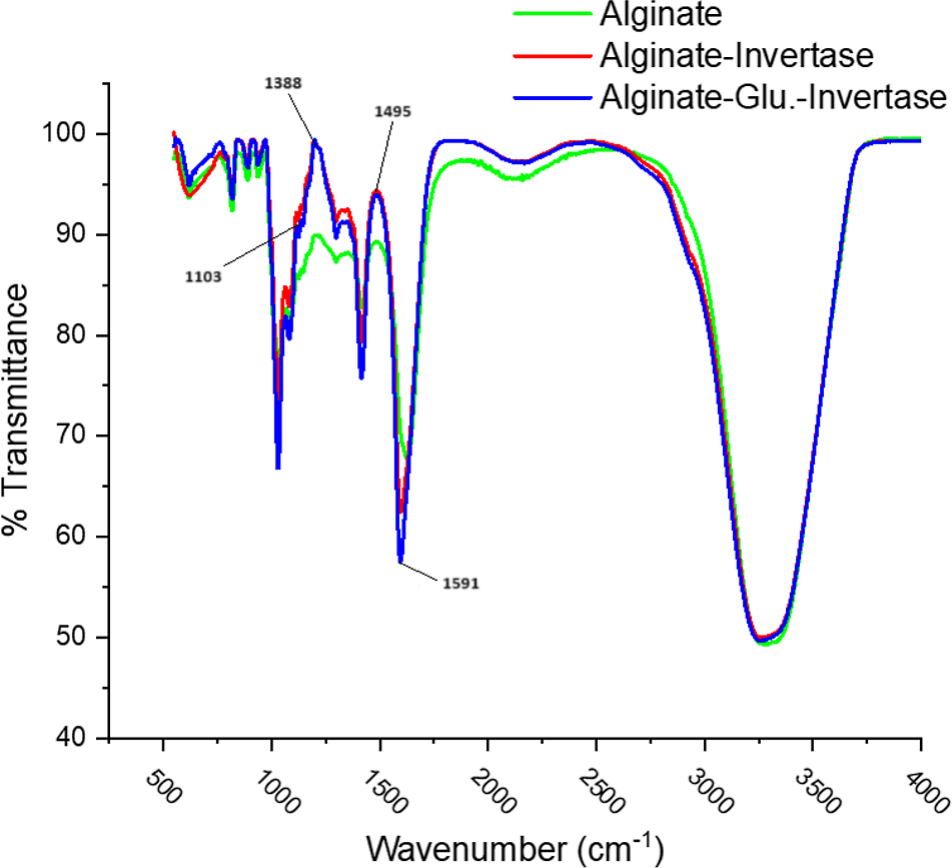
FTIR spectra of invertase, glutaraldehyde, and alginate beads.

The covalent binding of the invertase enzyme onto alginate beads with glutaraldehyde was investigated using Fourier transform infrared (FT-IR) spectroscopy to identify the functional groups present (Figure 3). The peak observed at 1591 cm^–1^ in the FT-IR spectrum of glutaraldehyde-activated alginate beads with immobilized invertase corresponds to the stretching vibration of the C=O groups. Additionally, significant vibrational modes of the enzyme were detected at 1103, 1388, and 1495 cm^–1^. These findings align with the results from the literature.^13,14^

In the literature, immobilization of the invertase isoform 1 (EINV1) was carried out on nanoclays.^7^ The effect of immobilization of invertase activity was investigated, and it was found that enzyme activity was significantly affected by the modification, ranging from 50 to 2200 U/g. The modified nanoclays showed that the immobilization process preserved the structure and catalytic properties of invertase, although the *K*_m_ values were slightly increased from 26 to 37 mM. Furthermore, immobilization resulted in improved thermal and storage stabilities of the enzyme. Modified beidellite nanoclays can serve as a suitable support for the immobilization of invertase, enabling its efficient use in batch reactors for sucrose hydrolysis.^7^ A low-cost and simple method was used to immobilize invertase onto magnetic diatomaceous earth nanoparticles (mDE-APTES-invertase), resulting in an enzyme with high sucrolytic activity.^37^ The immobilized invertase exhibited thermal stability at 35 °C for up to 60 min, retaining 85% of its activity, and storage stability for up to 120 days, retaining 80% of its activity. Additionally, mDE-APTES-invertase showed residual activities greater than 60 and 50% after short- and long-term reuse, respectively.

When these results were evaluated, it was observed that the pH and thermal stability of the immobilized enzyme increased despite the slight increase in *V*_max_ and *K*_m_ values. In addition, the enzyme became reusable. İmmobilized invertase can exhibit good catalytic activity at different process temperatures and pH values in fruit juice processes. At the end of the process, the immobilized enzyme is recovered from the environment and can be reused in subsequent processes, leading to a reduction in process costs.

### 3.4 Determination of Thermal and pH Stabilities of Purified and Immobilized Invertase

#### 3.4.1 Optimum pH and pH Stability

**Figure 4:**
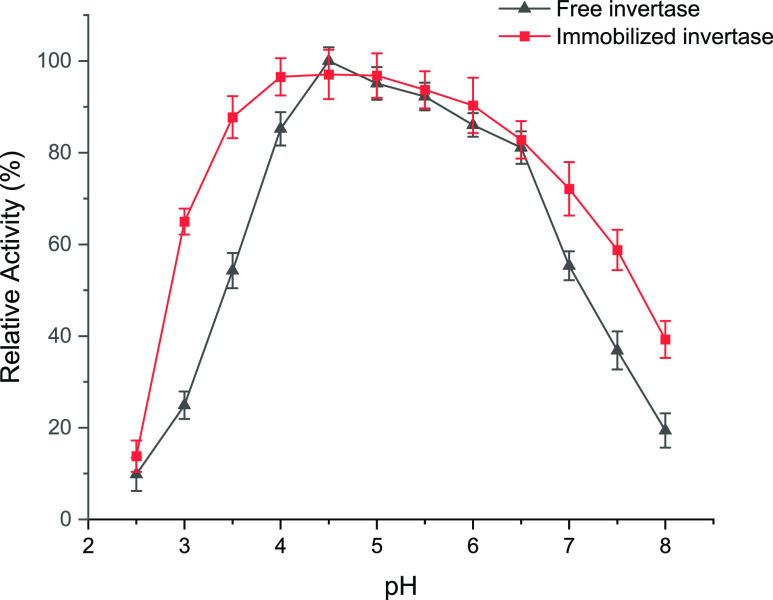
Effect of pH on the activity of purified invertase (free invertase) (blue line) and immobilized invertase (red line) in 50 mM acetate buffer in the pH range of pH 2.5 to 8.0.

The impact of pH on the activity of invertase was investigated by employing the DNS method at various pH levels ranging from 2.5 to 8.0 while maintaining a temperature of 55 °C. Figure 3 illustrates the pH activity curves of the free and immobilized enzyme. The outcomes indicated that the purified free enzyme had high activity in the range of pH 4.5 to pH 6.5. Figure 4 shows that the maximum activity for the immobilized enzyme was observed at about pH 5.0, and the immobilized enzyme maintains more than 80% of its relative activity in the range from pH 3.5 to 6.5. This result is consistent with the previous report that the optimum pH may shift significantly depending on the charge properties of the matrix. Additionally, immobilized invertase exhibited higher activity values for all pH values when compared to free enzyme, indicating improved operational conditions.

**Figure 5:**
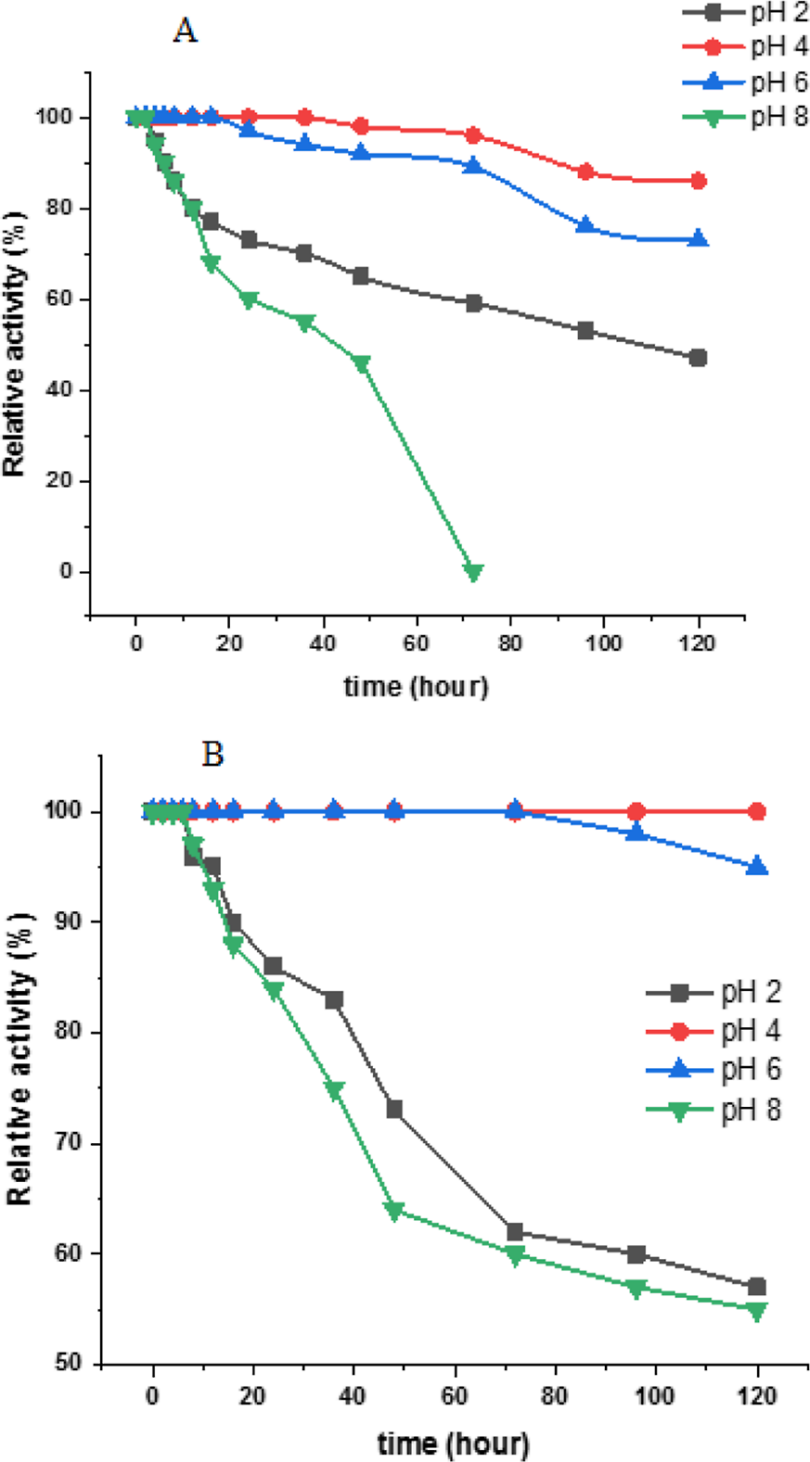
pH stability of free and immobilized invertase at pH 2.0, 4.0, 6.0, and 8.0. (A) pH stability of purified invertase, (B) pH stability of immobilized invertase.

Andjelković et al. purified the invertase of *S. cerevisiae*, modified it with beidellite nanoclay, immobilized the enzyme, and determined its optimum pH value to be 5.0 with only a slight shift.^7^ This change at pH 5.0 is thought to be due to the nature of the porous immobilization material used. Similarly, in our study, we found that the immobilized invertase enzyme showed a tendency to shift upward from pH 4.5 toward higher values after immobilization. Mansour and Dawoud reported that the optimum pH of invertase immobilized on Celite and polyacrylamide was found to be 4.5 at 60 °C.^38^ The pH stability of the purified invertase was evaluated by determining its activity at different pH values until 120 h of incubation (Figure 5A).

In another study, the invertase enzyme from Baker’s yeast was immobilized on a synthesized terpolymer membrane composed of *N*-vinylpyrrolidone, butyl acrylate, and *N*-(hydroxymethyl)acrylamide, utilizing a method of covalent bonding. The optimal pH for the unbound enzyme was identified as pH 5.0, while the immobilized enzyme exhibited an optimal pH of 7.0. The activity of the free enzyme exhibited a rapid decline when the pH deviated from 5.0. Conversely, the immobilized enzyme maintained a high level of activity within a pH range of 6.0 to 7.0. Remarkably, while the free enzyme lost all activity at pH 9.0, the immobilized enzyme remained active even at pH 11.0.^39^ Our study demonstrated that immobilized invertase exhibits greater pH stability. This finding highlights the superior pH versatility of the immobilized enzyme relative to that of its free form.

The purified enzyme showed good stability at its optimal pH values, with retention of about 50, 90, and 70% of its initial activity at pH 2.0, 4.0, and 6.0, respectively. Furthermore, the pH stabilities of the immobilized enzyme were investigated (Figure 5B). The immobilized enzyme was found to be more stable at pH 2.0 and 4.0 than at pH 6.0. The immobilized enzyme retained almost more than 60% of its initial activities after 120 h of incubation at the respective optimal pH values. These results indicate that immobilization of invertase into alginate beads significantly increased its pH stability.

In the literature, invertase from Baker’s yeast was immobilized on acid-activated montmorillonite clay (K-10) utilizing two distinct methodologies: adsorption and covalent binding.^40^ The free form of invertase demonstrated its peak enzymatic activity at pH 5.0. The process of immobilization resulted in an expanded pH profile from 4.0 to 7.0. Furthermore, the immobilization process conferred enhanced pH stability to the enzyme. At the enzyme’s optimum pH, the free form of invertase experienced a 35% reduction in activity after 300 min. In contrast, the immobilized forms managed to retain 90% of their initial activity.^40^ The results obtained from our study are parallel to those in the literature. The immobilized invertase has demonstrated quite a high pH stability.

#### 3.4.2 Optimum Temperature and Thermal Stability

**Figure 6:**
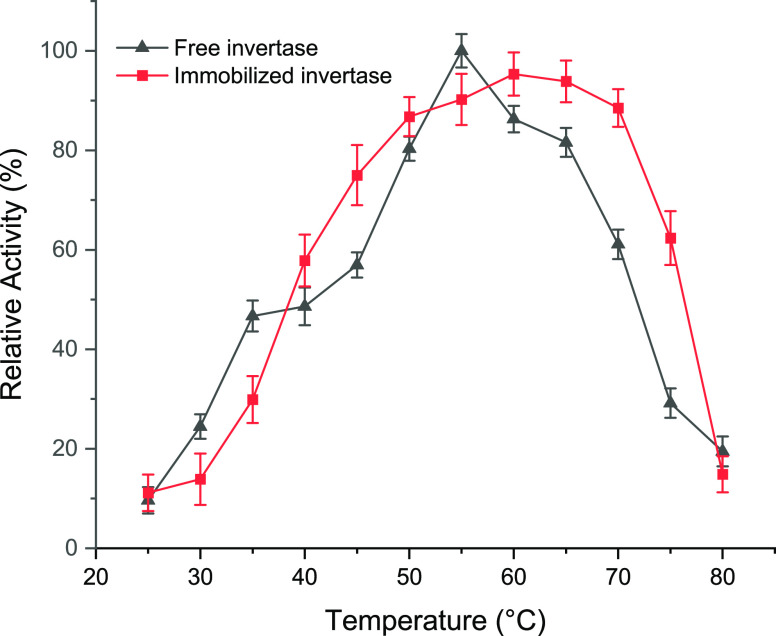
Relative activity for free and immobilized invertase at different temperatures (from 25 to 80 °C) under the optimum conditions.

The study investigated the effect of temperature on the activity and thermal stability of invertase. The activity of both free and immobilized enzymes was measured at different temperatures, ranging from 25 to 80 °C for the free enzyme and the immobilized enzyme. Results indicated that both free and immobilized enzymes showed broad temperature ranges for optimal activity, with the free enzyme reaching the maximum activity at 55 °C and the immobilized enzyme at 65 °C. Also, the immobilized invertase showed higher thermal stability than that of the free invertase at all temperatures from 25 to 80 °C (Figure 6).

Immobilization was found to enhance the operational conditions of invertase, with the temperature optima shifting to higher temperatures. For instance, the temperature optima of invertase shifted, and the immobilized enzyme maintained its high activity for an extended range from 50 to 70 °C. It is observed that the immobilized invertase can be used for the processes including high temperature levels.

A study in the literature reported that invertase from a yeast strain was immobilized on organomodified beidellite. Immobilization on beidellite clay resulted in a shift of the maximal values of initial activities from 55 °C toward 65 °C at pH 5.0.^7^ In another study, however, a method was developed to trap *S. cerevisiae* invertase within a supermacroporous polyacrylamide cryogel.^41^ The process resulted in a 74% activity yield. The immobilized invertase retained all of its initial activity for 30 days and 30 batch reactions. Immobilization did not affect the optimum temperature, which remained at 60 °C for both the free enzyme and the immobilized enzyme. However, the immobilized enzyme demonstrated greater stability than the free enzyme under conditions of high pH and temperature.^41^

Another study on invertase from *S. cerevisiae* showed that the thermal stability of the enzyme was investigated for three different forms: immobilized invertase on Celite, immobilized invertase on polyacrylamide, and free invertase.^38^ The results indicated that the order of decreasing stability was immobilized on Celite, immobilized on polyacrylamide, and soluble invertase. The activity of the enzyme decreased by 48.6, 67.5, and 87.9% for Celite, polyacrylamide, and soluble invertase, respectively, after being incubated at 70 °C for 30 min^38^

**Figure 7:**
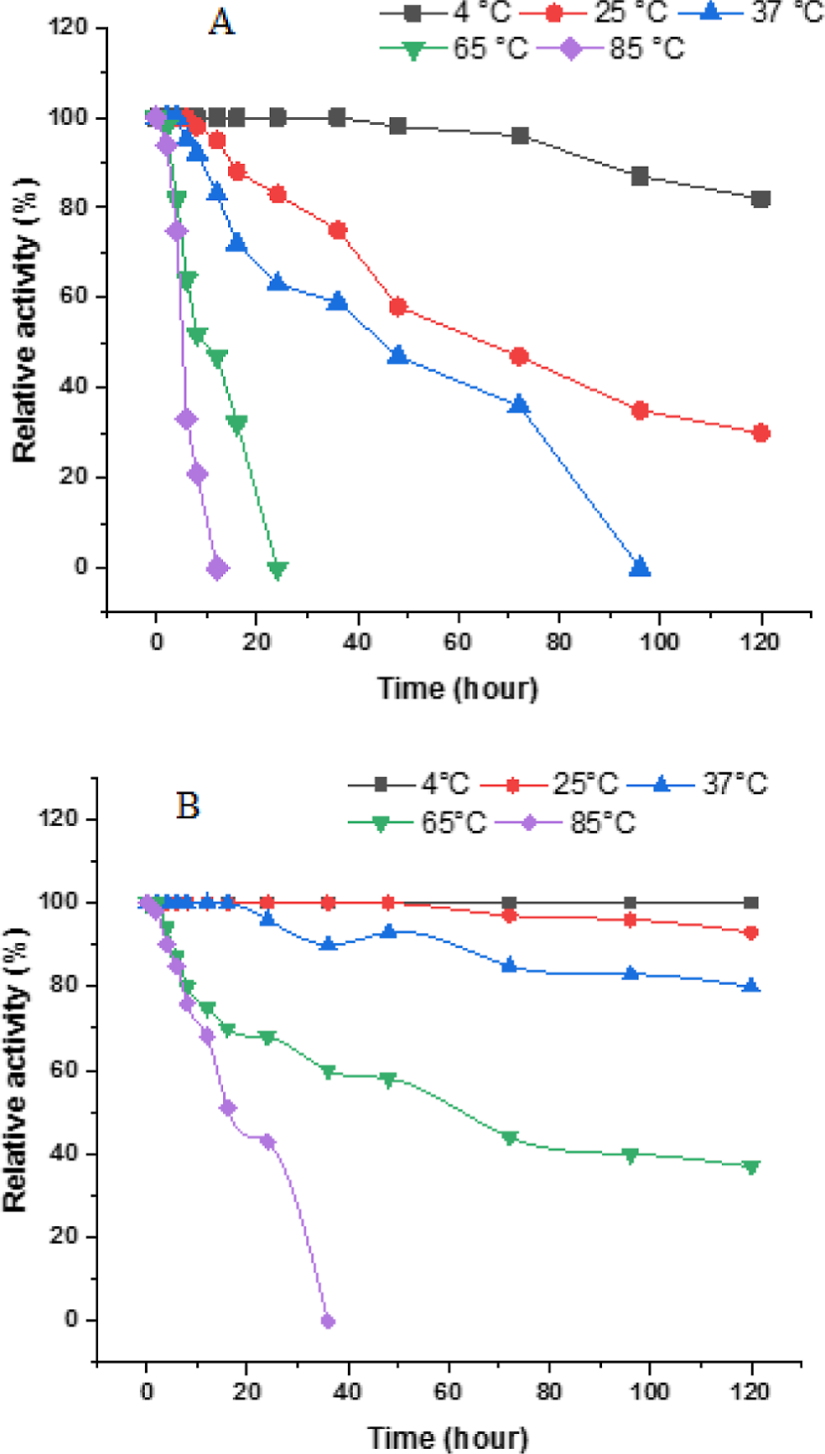
Thermal stability of invertase, (A) purified invertase, (B) immobilized invertase, and enzyme solutions were incubated at different temperatures (4, 25, 37, 65, and 85 °C) for varying durations in 50 mM acetate buffer at pH 5.0. The remaining activities of the enzyme were determined by comparing them with a standard assay mixture that contained nonincubated enzyme.

In this study, the thermal stability of both free and immobilized invertase was also tested at different temperatures (4, 25, 37, 65, and 85 °C). Results indicated that the free enzyme conserved almost 60% of its original activity after 36 h of incubation at 4, 25, and 37 °C, with almost all activity lost after incubation at 65 and 85 °C for 16 h (Figure 7A). In comparison, immobilized invertase into alginate beads preserved over 80% of their initial activities after 120 h of incubation at 4, 25 and 37 °C. After 24 h at 85 °C, the immobilized invertase still retains approximately 40% of its initial activity (Figure 7B). The data obtained after immobilization shows that the immobilized invertase enzyme can operate with high activity over a wider temperature range, and it is observed that its thermal stability has significantly increased. Based on the results, it is seen that the immobilized invertase enzyme is quite suitable for industrial applications.

### 3.5 Determination of the Kinetic Constant

It is necessary to elucidate the industrial performance of the immobilized invertase enzyme. For this reason, enzyme kinetic studies have been conducted. The *K*_m_ value, a key kinetic parameter, signifies the enzyme’s affinity for its substrate. It is defined as the substrate concentration at which the reaction rate is half of its maximum (*V*_max_/2).^42^ Essentially, it represents the substrate concentration at which half of the enzyme’s active sites are occupied. A lower *K*_m_ value indicates a high affinity of the enzyme for its substrate, meaning the enzyme reaches saturation and its maximum rate at a relatively low substrate concentration. Conversely, a high *K*_m_ value suggests a lower affinity, requiring a higher substrate concentration to saturate half of the enzyme’s active sites. The *V*_max_ value denotes the maximum velocity or speed of the reaction. At this maximum rate, all active sites of the enzyme are saturated with the substrate.^43^ To investigate the impact of concentration on the reaction rate of both free and immobilized enzymes, the reaction rate was determined at varying sucrose concentrations. A Lineweaver–Burk plot was used to compare the performance of the immobilized enzyme to that of the free enzyme.

The *K*_m_ value of the invertase was determined using a substrate range of 2.5 to 300 mM sucrose in 50 mM acetate buffer (pH 5.0). However, when industrial applications are considered, it is important to investigate how any observed differences between isoforms impact not only the stability and chemical reactivity of the enzyme but also their catalytic activity.

*K*_m_ and *V*_max_ values were determined by a Lineweaver–Burk plot as 1.27 mM and 434.78 U/mg protein for the purified enzyme and 1.54 mM and 513.97 U/mg protein for the immobilized enzyme into alginate beads, respectively. The *K*_m_ and *V*_max_ values of invertase increased slightly after immobilization.

β-d-fructofuranosidase from a novel fungus (Aspergillus sp.) was purified by ion-exchange chromatography.^33^ The substrate affinity of β-d-fructofuranosidase was evaluated through kinetic studies using d-sucrose as the substrate and analysis of the results using a Lineweaver–Burk plot. The obtained values of *K*_m_ and *V*_max_ were 0.85 mM and 47.62 U/mL, respectively, indicating a high affinity of the enzyme for the substrate.^33^ Another invertase isoform from *S. cerevisiae*, which was used in a substrate range that included sucrose concentrations from 2.5 to 300 mM in 50 mM acetate buffer at pH 4.50, had a *K*_m_ value of 25.6 mM.^7^

In another study conducted by Hassan and colleagues in 2019, a covalent immobilization of an enzyme called glucoamylase was performed onto a chemically activated k-carrageenan surface.^44^ They observed changes in the kinetic parameters of the enzyme. The apparent *K*_m_ of the immobilized enzyme (147.46 mM) was higher than that of the free enzyme (110 mM). This suggests that the immobilized enzyme has a lower affinity for its substrate.

The increase in the *K*_m_ value after immobilization could be due to the diffusion of the substrate into the alginate beads. While the immobilization process increases the enzyme stability, diffusion problems in enzyme–substrate interaction may occur due to the pores on the alginate beads.^45^ In other words, the change in *K*_m_ value was considered to be caused by electrostatic attraction between the carrier and the substrate.^46^ This situation can lead to an increase in the *K*_m_ value. Moreover, the three-dimensional structure of the enzyme within the alginate beads can be affected by changes in environmental conditions, resulting in changes in the conformation of the active site. This can also increase the *K*_m_ value. However, since the change in the *K*_m_ value is quite small, it is thought that the loss of activity after immobilization is low. Also, when compared with the *K*_m_ values determined for S. cerevisiae invertase in the literature, it is seen that the immobilized invertase used in this study has sufficient catalytic kinetic values.

### 3.6 Effect of Some Chemicals on the Enzyme Activity

The activity of many enzymes, including invertase, can be affected by the presence of specific metal ions. In particular, the activity of invertase may be influenced by calcium ions (Ca^2+^) and magnesium ions (Mg^2+^).^31^ These ions have the potential to interact with the enzyme, possibly altering its structure or its interaction with the substrate, leading to changes in the enzyme’s activity. However, the precise effect can vary and would necessitate experimental determination. It is also crucial to note that while certain metal ions can enhance enzyme activity, others may inhibit it.^31^

**Figure 8:**
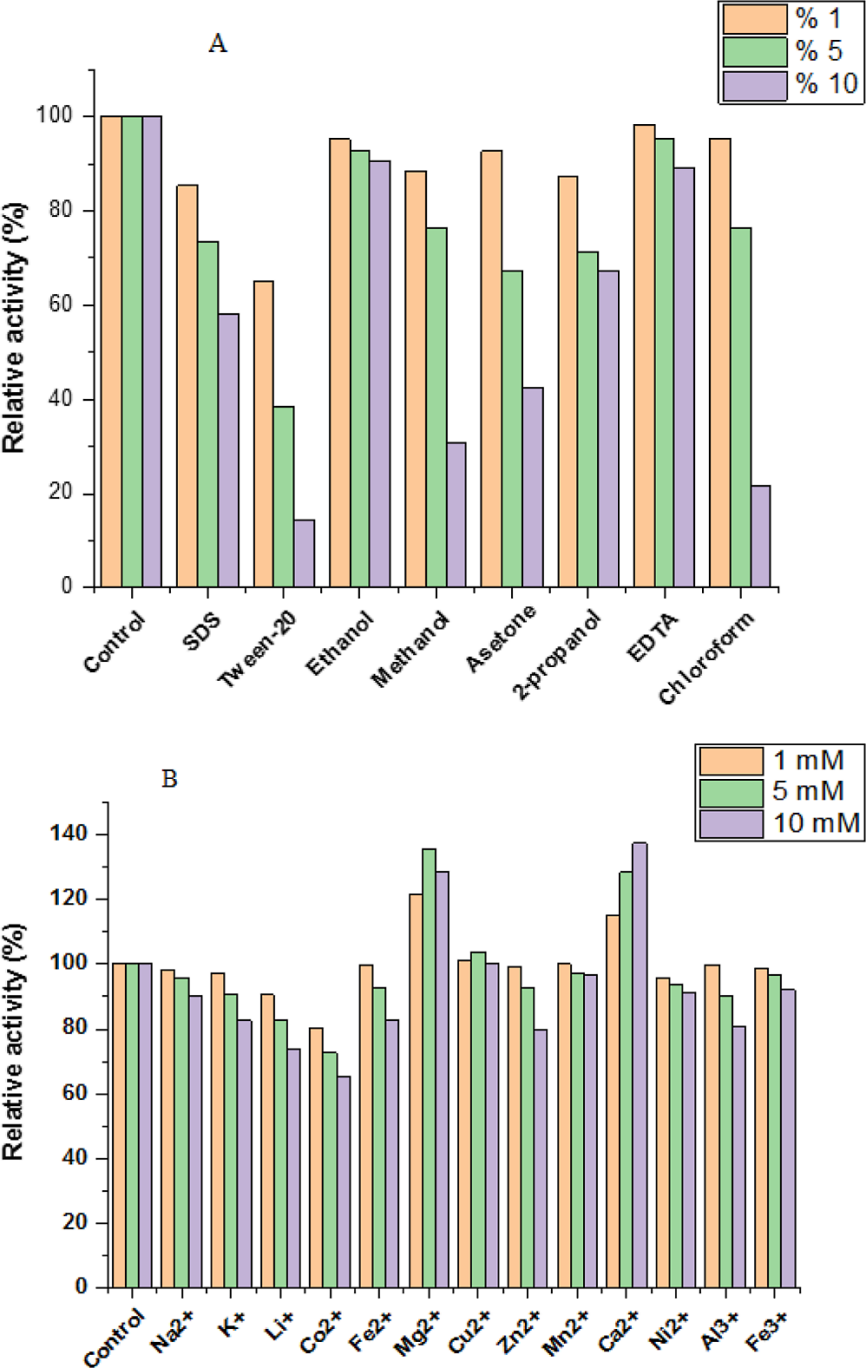
Effect of some chemicals on the immobilized invertase activity: (A) effect of organic chemicals and detergents, (B) effect of metal ions under the optimum conditions.

The impact of different metal ions on the immobilized invertase was examined in this study. Results indicated that Co^2+^ ions exhibited the inhibition of immobilized enzyme activity, while other metal ions showed slightly varying degrees of inhibitory effects (shown in Figure 8B). In addition, Ca^2+^ and Mg^2+^ ions have a positive effect on the immobilized invertase activity. The data obtained in Table 3 have shown parallel results with the effect of metal ions. In Table 3, the environments containing Mg^2+^ showed high invertase activity, which is seen as parallel to the positive effect of Mg^2+^ in metal ion effect studies. Considering the obtained data, it is thought that Mg^2+^ has a positive effect on the invertase activity.

Chemicals have the potential to modify the microenvironment and three-dimensional configuration of a protein, thereby influencing its activity in various manners. Similarly, research on invertase activity has indicated that its activity can be impacted in diverse ways by chemicals.^25^ The study also investigated the impact of various detergents (surfactants and possible chemical inhibitors) on the enzyme activity and found that the immobilized enzyme retained almost all of its original activity in the presence of 1, 5, and 10 mM of detergents. Conversely, 5 and 10% EDTA, ethanol, and 2-propanol slightly decreased the activity of the immobilized enzyme, while Tween-20 exhibited significant inhibition of the immobilized enzyme. The immobilized enzyme showed activity for all concentrations of detergents (Figure 8A).

In the literature, it has been reported that invertase, derived from *S. cerevisiae*, was studied in the presence of various surfactants such as triton X-100 (1%), polyethylene glycol (PEG), SDS, and Tween-20.^47^ The study found that as the concentration of these surfactants increased and the exposure time lengthened, the relative activity of invertase decreased. Interestingly, the study recorded the highest invertase activity, 35.88%, when the concentration of polyethylene glycol was at 1%. Conversely, the lowest invertase activity, 10.46%, was observed when the concentration of Triton X-100 was at 1%. These findings suggest that different surfactants can have varying effects on the activity of invertase, highlighting the complex interplay between enzyme activity and surfactant concentration.^47^

The heterologous expression of an invertase gene (GspInv) of Gongronella sp. in Komagataella pastoris was reported.^48^ The effects of metal ions on GspInv activity were examined. In the presence of Ca^2+^, the invertase activity increased to 111.9 ± 4.9% (1 mM), 112.7 ± 3.0% (5 mM), and 120.9 ± 3.7% (10 mM). Additionally, in this study, it was observed that the value of Mg^2+^ (1 mM) slightly increased the activity of the invertase enzyme (102.6 ± 1.8%). However, at concentrations of 5 mM Mg^2+^, the activity showed a partial decrease, observed as 90.7 ± 8.5%).^48^

This study demonstrated that when invertase was immobilized within calcium alginate beads, there was a notable increase in its activity in the presence of Mg^2+^ and Ca^2+^ ions. This observation aligns with the existing literature on the subject. Furthermore, it was discovered that the immobilized enzyme could maintain a portion of its activity even when potential surfactants, which are commonly used in industrial applications, were present. Therefore, it can be inferred that this immobilized invertase holds promise for potential utilization in industrial applications.

### 3.7 Reusability of Immobilized Enzyme

**Figure 9:**
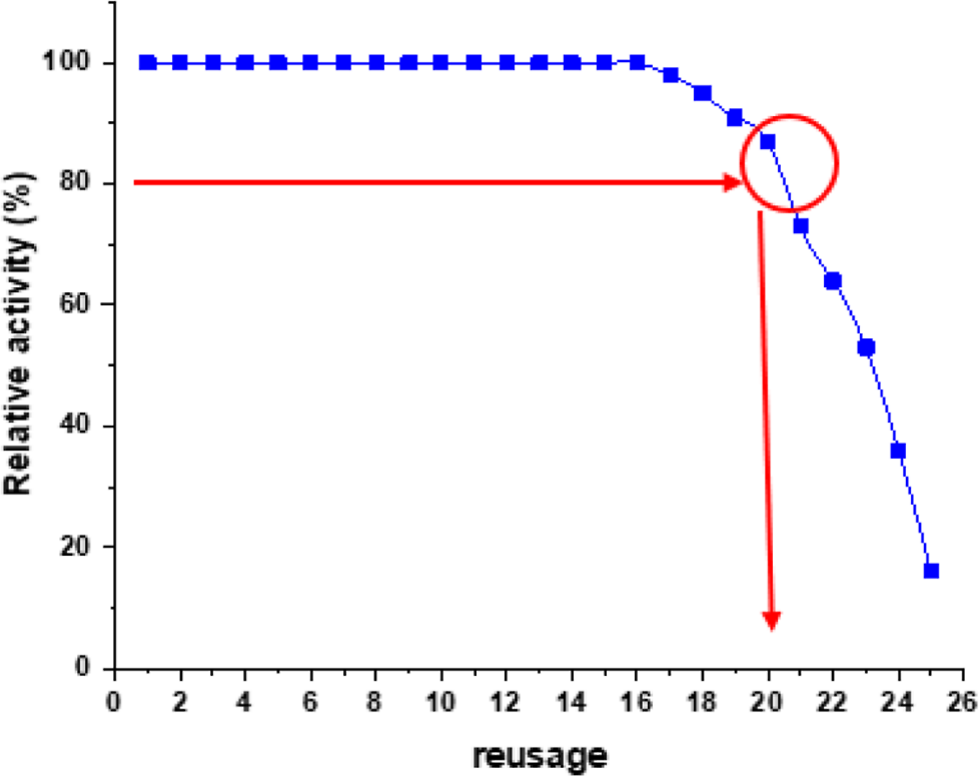
Reusability of the immobilized enzyme under optimum conditions.

For enzymes to be suitable for use in industrial applications, they must be able to endure harsh reaction conditions. One approach to ensure their stability under such circumstances is through the process of enzyme immobilization.^33^ The possibility of reusing immobilized invertase was examined by exposing it to 26 cycles under standard assay conditions, with the aim of determining its practicality for repeated use. There is 2 h period between each cycle. As illustrated in Figure 9, the immobilized enzyme retained 80% of its initial activity after 20 cycles.

In the literature, some studies showed that immobilized invertase retained 80% of total activity after 20 cycles.^37,38^ Also, they discovered that the immobilized enzyme was able to maintain most of its total activity over 20 consecutive cycles of use, demonstrating its potential for reuse. The researchers concluded that the process of enzyme immobilization, with its economic and biotechnical benefits and the potential for the enzyme to be reused multiple times, could lead to an increase in its applications across various industries.^37,38^ The findings in our study indicate that the immobilized invertases exhibit considerable stability, rendering them well-suited for use in continuous processes.

### 3.8 Hydrolysis of Sucrose by Immobilized Invertase

We evaluated the industrial efficiency of the immobilized invertase enzyme obtained from Type-1. The immobilized invertase (0.1 g) was added to a sucrose solution (1 L) (75% w/v), and the mixture was incubated at 55 °C for 16 h.

**Figure 10:**
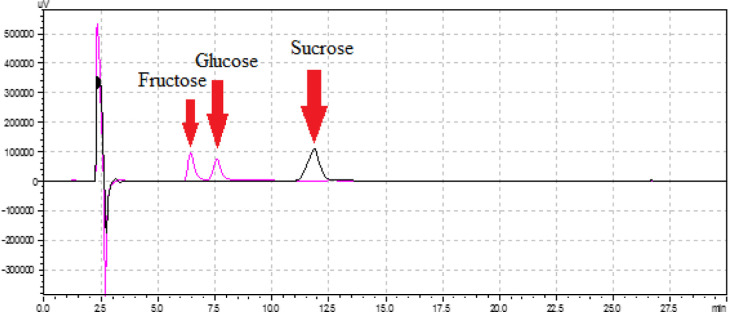
HPLC profile of sucrose without the immobilized invertase (black line) and with the immobilized invertase (purple line).

The breakdown of sucrose by immobilized invertase was monitored by using an HPLC system. As shown in Figure 10, sucrose exhibited an approximate retention time of 12 min, whereas the enzymatic conversion into glucose and fructose by the immobilized enzyme resulted in the observation of two peaks at 7.5 and 6.5 min, respectively. The HPLC profile from the experiment is parallel to the literature. The HPLC analysis showed similar retention time profile for fructose, glucose, and sucrose to the literature.^49^

Thus, it has been illuminated through the HPLC system that the immobilized invertase enzyme can be utilized in industrial fruit juice production, and in fruit juice manufacturing processes, the immobilized enzyme is capable of effectively breaking down sucrose into fructose and glucose.

## 4 Conclusions

This study has successfully demonstrated the commercial viability of using fruit waste, specifically pulp, to produce an invertase enzyme derived from *S. cerevisiae*. The research has shown that fruit pulp can serve as an effective and sustainable alternative to molasses, thereby offering a novel approach to repurposing fruit waste and reducing environmental impact.

The study found that carob pulp enhances the invertase activity considerably. The extracted enzyme is purified by ion-exchange column chromatography and immobilized by alginate beads. The immobilized invertase showed improved pH and thermal stability. The presence of Ca^2+^ and Mg^2+^ ions also boost the enzyme’s activity. The immobilized enzyme retained 80% of its total activity after 20 cycles, demonstrating its industrial efficiency and reusability.

Overall, this study establishes the commercial potential of fruit pulp as a valuable source for obtaining the invertase enzyme and highlights the benefits of harnessing fruit waste for enzyme production. Additionally, this study proposes a purification and immobilization process for the obtained invertase enzyme to enhance its industrial use. By implementing these innovative enzyme production strategies, industries can enhance efficiency, reduce their environmental footprint, and optimize resource utilization, thereby contributing to a more sustainable future.
