# Correction to “Industrial Approach to Invertase Production from Fruit Waste for Enhanced Efficiency and Conservation”

**DOI:** 10.1021/acsomega.4c08928

**Published:** 2024-11-13

**Authors:** Emre Dokuzparmak

During editorial processing, a recurring spelling error was introduced into the ASAP article, where the term “fig” (referring to the fruit) was erroneously changed to the word “Figure” (referring to images) throughout the manuscript. These spelling errors were corrected in the relevant sections.

In the Abstract: “By utilizing fruit pulp that incorporates mulberry, carob, **fig**, and grape pulp as a nutrient source, it is observed that the culture medium containing carob pulp exhibits the highest invertase activity.”

In the Introduction: “The pulp of **figs** is composed of various compounds, such as sugar, water, aromas, potassium, tartaric acid, and malic acid. However, it is worth noting that the total phenol, total flavonoid, and total anthocyanins of the frozen **figs** decrease significantly compared to fresh **figs**.”

In the Materials and Methods section, under the Materials subheading: “SDS-PAGE Sample Prep Kit was obtained from Thermo Fisher Scientific. Fruit pulps (mulberry, carob, **fig**, and grape pulp) were obtained from fruit juice factories in Turkey.”

In the Results and Discussion section: “To induce higher amounts of invertase enzyme from this strain, 13 different culture media were prepared, containing peptone, different sugars, and industrial waste materials such as mulberry, carob, **fig**, and grape pulp”.

In the Results and Discussion section: “As shown in Table 3, the media containing a combination of MgSO_4_, (NH_4_)2SO4, K_2_HPO_4_, and fruit pulp (carob pulp (M9) and **fig** pulp (M12)) showed higher invertase activity. In addition, the absence of MgSO_4_, (NH_4_)_2_SO_4_, and K_2_HPO_4_ in media (M1) resulted in low invertase activity. The medium containing carob pulp (M9) had the highest invertase activity (12.8 ± 0.77 U/mg protein), which was approximately 50% higher than that of the invertase activity (8.15 ± 0.78 U/mg protein) in M2 medium. The medium containing **fig** pulp (M12) also showed the second highest invertase activity (11.9 ± 0.99 U/mg protein). The results showed that the presence of industrial waste materials increased the invertase activity, indicating the potential of these waste materials to be used as a contribution to invertase production processes and gain commercial value.”

In Table 3: “**fig** pulp”.

